# Epidemiology of cough in relation to China

**DOI:** 10.1186/1745-9974-9-18

**Published:** 2013-07-08

**Authors:** Kefang Lai, Jiayu Pan, Ruchong Chen, Baojuan Liu, Wei Luo, Nanshan Zhong

**Affiliations:** 1 State Key Laboratory of Respiratory Diseases, 1st Affiliated Hospital, Guangzhou Medical College, Guangzhou, GZ, China

**Keywords:** Cough, Epidemiology, Etiology, Risk factor, Quality of life

## Abstract

Cough is one of the most common complaints for which patients seek medical attention. Misdiagnosis and mistreatment of cough exist commonly in China. The prevalence of acute cough caused by upper airway infection fluctuates between 9% and 64% in the community, for chronic cough, the prevalence >10% in most surveys, ranging from 7.2%-33%. The common causes of chronic cough are upper airway cough syndrome (previously called as post nasal drip syndrome [PNDS]), cough variant asthma (CVA), gastroesophageal reflux related cough (GERD) and eosinophilic bronchitis (EB). There is a regional discrepancy regarding the prevalence of common causes of cough and distribution of gender among China, UK, USA, the most common cause of chronic cough in China are CVA, followed by UACS, EB and atopic cough (AC), the male is almost equal to female in numbers in China. The risk factors for cough includes cold air, smoking, environmental pollutants, noxious substances and allergens, and unreasonable diet habits.

## Background

Cough is the most common complaint in outpatient clinics. Based on its duration, cough can be divided into three classifications: acute cough (<3wk), subacute cough (3-8wk), and chronic cough (>8wk). In the US, nearly 30 million people seek evaluation for cough at outpatient clinics every year [1]. Approximately 12% of people in Britain are bothered by chronic cough daily or weekly [2]. Chronic cough severely impairs their quality of life. Patients with chronic cough visited outpatient clinic six times on average [3]. It has been estimated that 360 million dollars are spent on over-the-counter (OCT) medications for chronic cough in the US every year, and the total amount of money spent on the treatment of cough globally is enormous (> 10 billion dollars) [2]. Although no surveys on economic burden caused by chronic cough have been conducted in China, we postulate that the situation is not better than that in America. In China, 80.82% patients were diagnosed with chronic bronchitis or laryngitis antibiotics are commonly used in 91.58% patients with chronic coughs [4]. This review will focus on four different aspects of cough, including the prevalence, etiology, risk factors, and influence on quality of life.

### Prevalence of cough

Acute cough is one of the most common symptoms in respiratory out patient clinic, the common cause is an acute upper airway infection. Therefore, to some extent, the prevalence of acute upper airway infection can represent the prevalence of acute cough. The prevalence of cough caused by upper airway viral infection fluctuates between 9% and 64% [5]. In patients having sore throat, 50% complain of cough [6].

Despite of the differences of surveyed population and criteria, many surveys on the prevalence of cough revealed a high incidence, which is commonly >10% (Table 1). Barbee et al. carried out a survey on the communities in Arizona and found out the prevalence of chronic cough was 18% [7]. The survey conducted in Italy showed that the incidence of chronic cough was 11.9% [8]. A survey in Switzerland reported that the prevalence of chronic cough in smokers was 3 times higher than non-smokers [9]. In southeast England, 16% of interviewees spent half of their time coughing, 13.2% of them reported productive cough, and over half of them (54%) smoked [10]; A cross-sectional international population survey conducted in 16 European countries involving 18277 interviewees revealed that the prevalence of chronic cough was 33%, and that of nocturnal cough was 30% [11]. However, this study did not differentiate between acute cough and chronic cough, and the high prevalence of nocturnal cough might have resulted from the design of some questions. The first cross-sectional survey on the prevalence of chronic cough was undertaken in West Yorkshire. A total of 36 general practices and nearly 4000 patients were involved in the study. After analyzing the bouts or spasms of coughing at a frequency between once a week and once or more a day in the last 2 months, Ford et al. reported that the prevalence of chronic cough was 12% [12]. A cough survey conducted in 1087 college students in South China revealed a prevalence of 10.9% with 7.6% acute cough and 3.3% chronic cough. No difference was found between male and female. The patients with chronic cough tend to present more likely as the sole or predominant symptom as compared with the patients with acute cough [13]. Since the college students have better health status and life style, we assumed the prevalence of cough could be much higher in the community.

**Table 1:** Prevalence of cough in different regions

**Surveys**	**Region**	**N**	**Definition of chronic cough**	**Prevalence(%)**
Barbee RA [7] (1991)	Arizona	1109	Having cough or phlegm on most days, as much as 3 months of the year , lasting for ≥ 2 years	18
	US			
Lundback B [14] (1991)	Norrbotten	6610		11
	Sweden			
Cullinan P [10] (1993)	southeast England	9077	Cough every day or one-half of the days in a year.	16
Ludviksdottir D [15] (1996)	Uppsala	623	Usually cough or production of phlegm in the morning during day or night in the winter	11
	Sweden			
Zemp E [9] (1999)	8 areas	9651	Usually cough during the day or at night, on most days for as much as 3 months each year.	Smoker: 9.2
	Switzerland			Nonsmoker:3.3
Coultas DB [16] (2001)	81 counties	5743	Having cough or phlegm on most days for ≥ 3 consecutive months per year	9.3
	US			
Janson C [11] (2001)	16 countries	18277	Usually cough or production of phlegm in the morning or during the day or night in the winter	33
	Europe			
Cerveri I [8] (2003)	9 areas	18000	Having cough and phlegm on most days for as much as 3 months per year and for at least 2 successive years	11.9
	Italy			
Carter ER [17] (2006)	Seattle	2397	Having a daily cough or phlegm as often as 3 months out of the year.	7.2
	US			
Ford AC [12] (2006)	Yorkshire	4003	Experienced bouts or spasms of coughing in the previous 2 months.	12
	UK			
Lai KF [13] (2006)	Guangzhou	1087	Cough lasting for > 8 weeks	10.9
	China			

Lai et al. investigated previous diagnosis and management of 558 outpatients with chronic cough in South China, found that the average number of visits for medical service was more than 20 for each patients [18]. More than 80% of the patients were misdiagnosed as "bronchitis, chronic bronchitis or pharyngitis". Antibiotics was given in about 90% of the patients. The results of the latest multicenter survey in China shows the incidence of using antibiotics previously was 69% in patients with chronic cough [19].

Cough is a most common symptom in children as well. Munyard and Bush reported that healthy children coughed 10 times everyday on average, the cough occurs most often in the daytime [20]. About 10%-22% of children aged 5–11 years had chronic cough in the absence of colds [21,22]. Some surveys demonstrated the prevalence of chronic cough in children 6–12 years of age was between 5% and 10% with a higher incidence in younger children [23,24]. Chronic cough occurred in 50% of children whose parents smoked [25].

### Etiology of cough

There are few reports on the spectrum of causes of acute cough and subacute cough. It is generally considered that the common cold is the most common cause of acute cough, followed by acute bronchitis. The causes of acute cough include acute exacerbation of chronic respiratory diseases (bronchiectasis, chronic bronchitis and COPD), noxious material and allergen-induced cough. Acute cough occasionally indicates some severe diseases such as pulmonary embolism, pneumonia and congestive heart failure, etc. Postinfectious cough is thought to be the most common cause of subacute cough. Kwon et al. evaluated 184 patients with subacute cough, found that postinfectious cough was in approximately 48% of the cases, followed by postnasal drip syndrome (33%) and cough variant asthma (16%) [26].

Irwin et al. [27] first set up an anatomic-diagnostic protocol for chronic cough in 1981, showed that the common causes of chronic cough were upper airway cough syndrome (UACS, termed as postnasal drip syndrome previously), bronchial asthma and gastroesophageal reflux disease (GERD). Some investigations in Europe and America have similar results on causes of chronic cough [28,29].

In 1989, Gibson et al. first reported 7 patients with eosinophilic bronchitis (EB), who presented with chronic cough, eosinophilia in induced sputum, normal airway responsiveness, and a good response to corticosteroids [30]. By adding the processing of induced sputum to the diagnostic algorithm of chronic cough, Brigthling et al. found that eosinophilic bronchitis was an important cause of chronic cough in 13% of patients [31]. Others studies from Australia, Korean, and Turkey also showed the proportion of EB to be 7-33% of patients presenting with chronic cough [32-34], In South China, Lai et al. reported that EB was the most common cause of chronic cough (22%), followed by PNDS (17%), CVA (14%), and GER (12%) [35]. Early studies in North China [36] and East China [37] showed EB was not as common as that in South China. This difference on causes may be due to various diagnosis proceeds. In a latest prospective, multicenters survey on causes of chronic cough in China, EB was identified to be one of the common causes in all regions, ranging from 9.4%-24.2% with a mean proportion of 17.2% [19]. EB has been formally proposed as one of common causes in the guideline on chronic cough by European Respiratory Society (ERS) [38], Chinese Medical Association [39], and American College of Chest Physicians (ACCP) [40]. Therefore, EB as a common cause of chronic cough has been recognized globally.

In the US or Europe, GERD is a common cause of chronic cough, however, GERD is found to be only a minor cause of chronic cough in Asia: 2% in Japan [41] and 4.6% in China [19]. The lower incidence of GERD-related cough might be related to the lower incidence of GERD in Asia as compared with the US or Europe [42]. In contrast to typical Western life style, the Asian dietary consists of high content of fiber, grains and vegetables. A diet rich in fiber has been reported to decrease the risk of cough [43].

Atopic cough (AC) was defined first by Fujimura in Japan as a bronchodilator-resistant dry cough associated with sputum eosinophilia or an atopic predisposition [44,45], and was reported being a common cause of chronic cough in Japan. AC reported in Japan overlap with EB in fact. The definition of AC in China is different from that of Japan since the patients with eosinophilia in induced sputum were excluded in China. Although the concept of AC remains controversial and is not widely accepted,many patients with atopic predisposition are seen in our clinics, and the cough can be relieved by corticosteroids or antihistamine therapy. Two regional surveys in Guangzhou and Shenyang, a Southern and a Northern region of China respectively, show a proportion of approximately 12% AC in patients with chronic cough [35,46]. Recently, a prospective, multicenter survey in China found that AC was identified in 13.2% of patients with chronic cough [19].

Idiopathic cough (or unexplained cough) would not be considered until a thorough diagnostic and treatment approach for the common causes of cough have been completed and uncommon causes are adequately evaluated [47]. The incidence of idiopathic cough is not uncommon in some studies [48,49]. In China, the unexplained cough accounted for 8.4% of the cases in a latest multicenter survey [19]. It is still unclear whether some unexplained cough (idiopathic cough) represents a single entity or includes certain undiscovered causes of chronic cough. Those idiopathic cough are more common in middle age women and perimenopause women, characteristiced by being hypersensitive to smoke, dust, cold air and other stimulants, increased cough sensitivity, no causes could be identified in spite of many investigation. Recently, a new single discrete clinical entity termed as ’cough hypersensitivity syndrome’ was introduced. It was postulated that the new term would focus on the problem on the cough symptomatology and lead to a better understanding of the mechanisms of cough sensitization [50,51].

Although epidemiological surveys in the community show a high incidence of chronic bronchitis, the hospital-based studies on the etiology of chronic cough have shown that the proportion of these patients is only 4%-7% [27,28] (Table 2).

**Table 2:** Causes of chronic cough in different regions

	**N**	**Asthma**	**PNDS**	**GERC**	**EB**	**Other causes**
**USA**						
Irwin RS [27] (1980)	49	25%	29%	10%		CB 12%
Poe [52] (1989)	139	35%	26%	5%	/	CB 7%
Irwin RS [28] (1990)	102	24%	41%	21%	/	CB 5%
Mello [29] (1996)	88	14%	38%	40%	/	/
**UK**						
Brightling CE [31] (1999)	91	18%	24%	8%	13%	
Birring SS [53] (2004)	236	17%	12%	15%	7%	PIC 7%
Kastelik JA [54] (2005)	131	24%	6%	22%	/	PIC 8%
**Australia**						
Carney IK [32] (1997)	30	23%	73%	93%	10%	ACEI 23%
**Turkey**						
Ayik S [34] (2003)	36	3%	22%	22%	33%	PIC 6%
**Japan**						
Fujimura M [41] (2005)	176	36%	18%*	2%	/	AC 29% #
**Korea**						
Joo JH [33] (2002)	92	16%	33%	/	12%	CB 15%
**China**						
Yang ZM [37] (2005 Shanghai)	105	51.4%	26.7%	1.9%	5.7%	PIC 8.5%
Lai KF [35] (2006 Guangzhou)	194	14%	17%	12%	22%	AC 12%
Lu GL [36] (2009 Beijing)	123	33.3%	24.4%	20.3%	4.9%	AC 3.3%
Lai KF [19] (2012)	704	32.6%	18.6%	4.6%	17.2	AC 13.2%

Abbreviations: *PNDS* Post-nasal drip syndrome, *GERC* Gastroesophageal reflux-related cough, *EB* Eosinophilic bronchitis, *AC* Atopic cough, *PIC* Post-infectious cough, *CB* chronic bronchitis, *ACEI* ACEI-related cough.

# The definition of atopic cough in Japan is somewhat different from that in China.

Angiotensin converting enzyme inhibitor (ACEI)-induced cough should be noted predictively in the elderly. Because the incidence of cardiovascular disease is associated with age, elderly people are might expected to have ACEI-induced cough more frequently [55].

The common causes of chronic cough in children vary according to different age groups [56]. Acute respiratory infection and protracted bacterial bronchitis are the most common causes for infant and early children [57]. Allergy and asthma become predominant causes during the school years [58]. Foreign body aspiration is much more common in infant than adults. Gastroesophageal reflux during infancy is a physiological phenomenon with an occurrence rate of 40% to 65%, reaching a peak in infants of 1 to 4 months, and this eases after age of 1 year. It is debatable whether GERD is a common cause of chronic cough in children. Chang et al. found a low incidence of GERD related to chronic cough in children [24]. However, a high incidence of GERD related chronic cough was reported in another study [58]. There are few reports concerning the incidence of EB in children with chronic cough. In China, the epidemiological surveys concerning chronic cough in children are lacking. Zhao et al. analyzed the causes of chronic solitary cough in 50 children aged 6–12 years, finding that 20 were PNDs 17 CVA, 5 postinfectious cough, 4 psychogenic cough, 1 primary gastroesophageal reflux disease and 2 EB [59].

### Risk factors for cough

Cold air, cigarette smoking, allergens, environmental pollutants, various noxious substances, poor diet habit and female gender may contribute to cough.

#### Cold air

The winter season is the peak period of incidence for respiratory diseases [60]. It might due to direct the simulating effect of cold air on airway mucosa and the cough reflex. In addition, the lower temperature increases the replication and spread of respiratory viruses. A long-life cohort study from 1998 to 2007 observed the seasonal variation of cough and showed that the proportion of subjects reporting cough was significantly higher in winter and fall, than in other seasons. In the winter season, levels of elemental carbon and NO2 in ambient air were significantly are associated with cough [61].

#### Cigarette smoking and exposure to tobacco smoke

Both cigarette smoking and exposure to tobacco smoke are risk factors for cough, including dry cough, productive and nocturnal cough [9]. Regular smokers have a much higher prevalence of chronic cough than never smokers and ex-smokers [7,9], whereas smokers generally disregard cough and refuse to seek medical assistance. In survey of Charlton et al., definite link was found between parental smoking in the home and increasing risk of coughs in young children [25].

#### Diet habits

Poor dietary habits such as long-term fat-diet and excessive stimulating food or coffee may induce relaxation of the lower esophageal sphincter, and lead to gastroesophageal reflux [62]. Compared with Asia, a higher prevalence of GERD (10-22%) [27,28,53,54]is found in Europe and Unite States as compared with Asia (2-12% ) [19,35,41], which may be related to long-term high fat-diet and coffee intake in Europe and America. The effect of lifestyle modification (including losing weight and low fat-diet) on decreasing the prevalence of GERD has been noted [63].

#### Environmental pollution

Exposure to increasing particulate matter (PM10) is associated with increased respiratory symptoms such as cough, phlegm and sore throat, and is likely to induce or worsen cough [64]. It was noted that PM10 increased the odds of cough and phlegm in school students aged 11–20 years old in Hong Kong [65]. A cohort study in Switzerland demonstrated that the prevalence of cough decreased with a fall of ambient PM10 concentration [66]. Chronic cough was more common among those subjects living close to heavy traffic compared to those not living close to heavy traffic [67]. An Italian study indicated that an increased prevalence of cough in females was due to air pollution [68]. Excessive exposure to nitrogen dioxide was also found to be a risk factor for cough [9]. In the heavy industrial province of Northeastern China, the high level of outdoor air pollution, such as TSP, SO_2_ and NO_2_ were positively associated with the increasing prevalence of chronic cough in children [69].

#### Allergen

There is evidence that allergen is a potential cough-related factor. Gehring and co-workers noted that indoor exposure to Dermatophagoides farinaeantigen and cat antigen increase the risk of nocturnal cough [70]. Allergic diseases such as EB and CVA might be induced due to exposure to pollen and dust mite, as well as occupational exposure to chemical agents [71-73].

#### Gender

Among patients attending specialist cough clinics, females exceed males in number [74]. ACEIs cause cough in 5–20% of patients who receive them, and females are more susceptible than males [75]. Studies have shown that chronic persistent nonproductive cough is more frequent in females [44,76]. Cough affects females more often than males, and females are more easily troubled by symptoms of cough. The cough threshold is lower in females than in males, illustrating that the cough sensitivity is heightened in females [77,78]. This gender difference of cough is not very obvious in China, the numbers of male is almost equal to female [4,13,35]. The mechanism underlying the gender differences in cough is still unclear, and additional research is needed to better understand gender differences in coughs.

### The influences of cough on quality of life

Chronic cough can lead to physiological disorder, psychological stress and disrupt social life. Complications induced by cough involve the respiratory, cardiovascular, digestive, genitourinary, musculoskeletal and nervous systems. An increasing prevalence of complications is in line with an aggravating and prolonged cough [79]. Thoracoabdominal pain caused by the damage of muscle fibers, as well as hoarseness due to vocal cords damage, are common in clinic. Many women with chronic cough experience urinary incontinence [80]. In China, Yang et.al reported that chronic cough seriously interfered with the patient's quality of life, which shows no gender difference. However, women have urinary incontinence with a proportion of about 50% [81].

Chronic cough impact seriously quality of life on physiological, psychological and social aspects. Questionnaires are used to comprehensively assess impact of cough on quality of life, such as Cough-Specific Quality of Life Questionnaire (CQLQ) [82], Leicester Cough Questionnaire(LCQ) [83] and Chronic Cough Impact Questionnaire (CCIQ) [84]. In addition to physiological complications, patients with chronic cough experience psychological imbalance and social predicament. Patients suffer from embarrassing emotion caused by prolonging cough, they feel anxious about the cough and prejudices from others, which may aggravate their psychological condition [82]. Among patients with chronic cough in New York, 53% of them experience depression, indicating that depression is a common incidental symptom [85]. Psychological symptoms are often relieved with inmprovement of chronic cough [84]. A survey in Guangzhou showed that patients with an average duration of 4 years of cough are also troubled with mental and social problems, one-half of patients feel embarrassed and annoyed, and greater than one-quarter feel upset and even depressed [86].

## Conclusion

Cough is one of the most common complaints for which patients seek medical attention. Health-care expenditure for managing cough is substantial and quality of life can be severely affected**.** The prevalence of acute cough is 9 to 64% in the community. The prevalence of chronic cough is >10% in most countries or regions, ranging from 7.2%-33%. The common causes of chronic cough are upper airway cough syndrome, cough variant asthma, gastroesophageal reflux-related cough and eosinophilic bronchitis. There are regional discrepancies about the common causes of chronic cough, gastroesophageal reflux-related cough in China and Japan is not so common as in Europe and America, and atopic cough is more common in China and Japan. The risk factors of cough includes cold air, smoking, environmental pollutants, noxious substances and allergens, and unhealthy dietary habits.

## Competing interests

The authors declare that they have no competing interests.

## Authors’ contributions

NSZ and KFL conceived the study and set up the task groups of the authors. All authors conducted the literature review. KFL, JYP, RCC, BJL, and WL created the draft of the paper. All authors read and approved the final manuscript.

## Authors’ information

KFL:M.D., Ph.D. Professor of Internal Medicine, State Key Laboratory of Respiratory Diseases, 1st Affiliated Hospital of Guangzhou Medical College, and Ph.D. tutor of respiratory medicine. JYP: M.D., State Key Laboratory of Respiratory Diseases, 1st Affiliated Hospital of Guangzhou Medical College. RCC: M.D., Ph.D. State Key Laboratory of Respiratory Diseases, 1st Affiliated Hospital of Guangzhou Medical College. BJL: M.D., State Key Laboratory of Respiratory Diseases 1st Affiliated Hospital of Guangzhou Medical College. WL: Master’s degree in immunology, Senior Research Fellow, State Key Laboratory of Respiratory Diseases, 1st Affiliated Hospital of Guangzhou Medical College. NSZ: Academician of Chinese Academy of Engineering, Professor of Internal Medicine, State Key Laboratory of Respiratory Diseases, 1st Affiliated Hospital of Guangzhou Medical College, and Ph.D. tutor of respiratory medicine.
